# Serotonergic modulation of the activity of GLP-1 producing neurons in the nucleus of the solitary tract in mouse

**DOI:** 10.1016/j.molmet.2017.06.002

**Published:** 2017-06-07

**Authors:** Marie K. Holt, Ida J. Llewellyn-Smith, Frank Reimann, Fiona M. Gribble, Stefan Trapp

**Affiliations:** 1 Centre for Cardiovascular and Metabolic Neuroscience, Department of Neuroscience, Physiology & Pharmacology, University College London, London, WC1E 6BT, UK; 2 Cardiovascular Medicine, Human Physiology and Centre for Neuroscience, Flinders University, Bedford Park, SA 5042, Australia; 3 Cambridge Institute for Medical Research, University of Cambridge, Addenbrooke's Hospital, Hills Road, Cambridge, CB2 0QQ, UK

**Keywords:** Serotonin, Preproglucagon, GCaMP, Dendritic calcium, NTS, 5-HT, 5-hydroxytryptamine, CCK-8, cholecystokinin-8, CNS, central nervous system, GLP-1, glucagon-like peptide-1, HPA, hypothalamic–pituitary–adrenal, IRT, intermediate reticular nucleus, NTS, nucleus tractus solitarius, PBN, parabrachial nucleus, PPG, preproglucagon, YFP, yellow fluorescent protein

## Abstract

**Objective:**

Glucagon-like peptide-1 (GLP-1) and 5-HT are potent regulators of food intake within the brain. GLP-1 is expressed by preproglucagon (PPG) neurons in the nucleus tractus solitarius (NTS). We have previously shown that PPG neurons innervate 5-HT neurons in the ventral brainstem. Here, we investigate whether PPG neurons receive serotonergic input and respond to 5-HT.

**Methods:**

We employed immunohistochemistry to reveal serotonergic innervation of PPG neurons. We investigated the responsiveness of PPG neurons to 5-HT using *in vitro* Ca^2+^ imaging in brainstem slices from transgenic mice expressing the Ca^2+^ indicator, GCaMP3, in PPG neurons, and cell-attached patch-clamp recordings.

**Results:**

Close appositions from 5-HT-immunoreactive axons occurred on many PPG neurons. Application of 20 μM 5-HT produced robust Ca^2+^ responses in NTS PPG dendrites but little change in somata. Dendritic Ca^2+^ spikes were concentration-dependent (2, 20, and 200 μM) and unaffected by blockade of glutamatergic transmission, suggesting 5-HT receptors on PPG neurons. Neither activation nor blockade of 5-HT_3_ receptors affected [Ca^2+^]_i_. In contrast, inhibition of 5-HT_2_ receptors attenuated increases in intracellular Ca^2+^ and 5-HT_2C_ receptor activation produced Ca^2+^ spikes. Patch-clamp recordings revealed that 44% of cells decreased their firing rate under 5-HT, an effect blocked by 5-HT_1A_ receptor antagonism.

**Conclusions:**

PPG neurons respond directly to 5-HT with a 5-HT_2C_ receptor-dependent increase in dendritic [Ca^2+^]_i_. Electrical responses to 5-HT revealed additional inhibitory effects due to somatic 5-HT_1A_ receptors. Reciprocal innervation between 5-HT and PPG neurons suggests that the coordinated activity of these brainstem neurons may play a role in the regulation of food intake.

## Highlights

• Brainstem PPG neurons receive close appositions from 5-HT-containing axons.
• 5-HT activates NTS PPG dendrites directly via 5-HT_2_ receptors.
• 5-HT inhibits a subset of somata via 5-HT_1A_ receptors.
• Activation of 5-HT_3_ receptors does not affect PPG cell [Ca^2+^]_i_.
• 5-HT_2C_ receptor activation induces spatially confined Ca^2+^ spikes in PPG neurons.

## 1 Introduction

Glucagon-like peptide-1 (GLP-1) is an incretin best known for its role in glucose homeostasis and appetite regulation [1]. Activation of brain GLP-1 receptors also increases heart rate and blood pressure, induces nausea, and activates the hypothalamic–pituitary–adrenal (HPA) axis in both humans and rodents [2], [3], [4], [5]. Along with reduced appetite, activation of the sympathetic nervous system and the HPA axis are both symptoms of stress; and stress-induced hypophagia has recently been shown to depend partly on activation of brain GLP-1 receptors [6], [7], [8].

Within the brain, GLP-1 is produced by preproglucagon (PPG) neurons in the lower brainstem [9], [10], [11], [12]. These neurons are ideally situated to receive information about homeostatic stress and energy balance from the rest of the body. Gastric distention and cholecystokinin, both peripheral signals of satiety, induce expression of the immediate early gene *c-fos* in GLP-1-expressing neurons, suggesting PPG neurons could play a role in relaying peripheral satiety signals [13], [14]. Indeed, chemogenetic activation of PPG neurons has recently been shown to induce hypophagia and body weight loss, suggesting these neurons have the potential to regulate food intake [15]. PPG neurons project from the nucleus of the solitary tract (NTS) and intermediate reticular nucleus (IRT) to autonomic control centers throughout the central nervous system, including sympathetic preganglionic neurons in the spinal cord [9], [16]. Many of these nuclei have been shown to express GLP-1 receptors [10], [17], and injection of GLP-1 or its analogs into brain tissue or ventricles produces effects on food intake, thermogenesis, and cardiovascular function [18], [19], [20]. Although it seems likely that PPG neurons are the source of GLP-1 released in the brain, the precise physiological role and cellular regulation of the activity of PPG neurons is still under intense discussion [21].

Serotonin (5-hydroxytryptamine; 5-HT) is another key neurotransmitter involved in both regulation of stress and anxiety and control of food intake [22], [23], [24]. 5-HT serves complex roles centrally, reflected in a diverse range of 5-HT receptor subtypes and widespread distribution of 5-HT receptor-expressing neurons within the brain. Receptors are expressed postsynaptically on both dendrites and somata and presynaptically on synaptic terminals and can be either inhibitory or excitatory. A variety of 5-HT receptor subtypes have been implicated in the regulation of food intake, including 5-HT_3_ [25], 5-HT_2C_ [26], 5-HT_1A_ [27], and 5-HT_1B_ [28]. To facilitate development of new obesity treatments, it is crucial to understand the underlying circuitry and the potential interactions with other anorexigenic and anxiogenic systems such as the brain GLP-1 system. 5-HT_2C_ receptors are important for control of food intake and loss-of-function mutation of the 5-HT_2C_ receptor leads to increased food intake, obesity and impaired glucoregulation [29], [30]. Interestingly, the food intake-suppressing effect of GLP-1 administered intraperitoneally was abolished in a mouse lacking 5-HT_2C_ receptors, suggesting a link between GLP-1 and serotonergic regulation of food intake [31], [32].

PPG neurons heavily innervate serotonergic neurons in the caudal raphe nuclei [33]; but it is currently unclear whether PPG neurons in turn receive serotonergic input. Patch-clamp electrophysiology demonstrated that PPG neurons are activated by the satiety hormones leptin and cholecystokinin-8 (CCK-8) as well as by glutamate and adrenaline [34], [35]. Although patch-clamp electrophysiology provides a highly sensitive method of characterizing the electrical properties of individual neurons, it is limited to recording from single cells. In contrast, genetically encoded Ca^2+^ indicators allow for the monitoring of the activity of entire functionally distinct populations of neurons [36].

In this study, we have investigated the cellular effects of 5-HT on PPG neurons, as well as the innervation that underlies these effects. We found that 5-HT-immunoreactive axons closely apposed PPG cell bodies and dendrites in the NTS. Using transgenic mice expressing the GFP-based Ca^2+^ sensor GCaMP3 selectively in the PPG neurons [37], we characterized the responses to 5-HT of both PPG cell bodies and dendrites [38]. In addition, cell-attached patch-clamp recordings allowed us to determine how 5-HT affected the action potential firing patterns of PPG neurons. 5-HT modulated PPG neuronal activity with different outcomes in dendrites and somata. Dendritic activation by 5-HT was dependent on 5-HT_2_ receptors and 5-HT_2C_ receptor activation was sufficient to elicit dendritic Ca^2+^ rises in PPG neurons. Interestingly, the Ca^2+^ changes in PPG neurons were compartmentalized with dendritic spikes failing to propagate to somata. In addition, about half of PPG neurons were found to decrease firing rate upon application of 5-HT, an effect that was abolished by blockade of 5-HT_1A_ receptors. These data suggest a multifaceted link between 5-HT and central GLP-1, which is tightly regulated through excitatory or inhibitory responses to 5-HT occurring within different subcellular compartments of PPG neurons.

## 2 Materials and methods

### 2.1 Transgenic animals

Two transgenic mouse strains were used in this study. The GLU-124 Venus YFP strain (YFP-PPG) [9], [39], which expresses YFP in PPG cells, was used for the immunohistochemical experiments. Mice expressing GCaMP3 in PPG neurons were obtained by crossing transgenic mice expressing Cre recombinase under the control of the glucagon promoter (GLU-Cre12) [40] with a ROSA26-lox-stop-lox-GCaMP3 reporter mouse as described earlier (GCaMP3-PPG) [37]. Mice were bred as homozygotes and kept on a 12 h light: 12 h dark cycle with *ad libitum* access to food and water. All experiments were carried out in accordance with the U.K. Animals (Scientific Procedures) Act, 1986, with appropriate ethical approval.

### 2.2 Immunohistochemistry

We used a total of three male and three female adult YFP-PPG mice. They were perfused at 12–16 weeks after birth and the tissue was processed as described earlier [33]. Both primary antibodies used here have been characterized previously in our laboratory [9], [33] and exhibit specificity.

Briefly, the medullas of YFP-PPG mice were blocked without the use of a brain matrix, infiltrated with sucrose and cut into three series of transverse 30 μm cryostat sections. Sections were first washed 3 × 10 min in 10 mM Tris, 0.9% NaCl, 0.05% thimerosal in 10 mM phosphate buffer, pH 7.4, (TPBS) containing 0.3% Triton X-100, and then exposed to TPBS-Triton containing 10% normal horse serum (NHS) for at least 30 min. Diluents were TPBS-Triton containing 10% NHS for primary antibodies; TPBS-Triton containing 1% NHS, for secondary antibodies; and TPBS-Triton, for avidin-horseradish peroxidase. Immunohistochemistry was done at room temperature on a shaker and washes were in TPBS for 3 × 10 min after each exposure to an immunoreagent.

Antigens were localized with our standard avidin-biotin-peroxidase protocol and either black or brown diaminobenzidine (DAB) reaction products. Sections that had been washed in TPBS-Triton and exposed to 10% NHS as above were transferred into 1:5000 rabbit anti-5-HT (Catalog #8250-0009, Lot #23073052; Biogenesis, Poole UK) for 2–3 days. After washing, sections were incubated overnight in a biotinylated donkey anti-rabbit immunoglobulin (Ig; 1:500; Jackson ImmunoResearch, West Grove PA) followed by a 4–6 h incubation in ExtrAvidin-peroxidase (1:1500; Sigma–Aldrich, St Louis MO, USA). A cobalt + nickel-intensified DAB reaction using peroxide generated by glucose oxidase [41] stained structures immunoreactive for 5-HT black. After 5-HT had been localized, the sections were washed and underwent another blocking step in NHS. Then the immunohistochemical protocol above was repeated to localize YFP with 1:50,000 chicken anti-GFP (Catalog #ab13970, Lot #623923; Abcam, Cambridge, UK). YFP-immunoreactive structures were stained brown with an imidazole-intensified DAB reaction in which peroxide production was catalyzed by glucose oxidase [41].

We examined stained sections from the spinomedullary junction to the rostral end of the area postrema with an Olympus BH-2 brightfield microscope equipped with a SPOT color camera (Diagnostic Instruments Inc., Sterling Heights, MI, USA). Images were captured as TIFF files using SPOT software v5.2 and exported to Adobe PhotoShop for adjustment of sharpness, brightness, and contrast. Montages of micrographs and plates were prepared with PhotoShop. Using an ×100 oil immersion lens, we quantified close appositions from 5-HT-immunoreactive axon terminals onto YFP-immunoreactive cell bodies and dendrites in one female and two male mice.

GCaMP3 immunoreactivity was detected with an antibody raised against green fluorescent protein (GFP; Catalog #AB13970, lot #623923; Abcam, Cambridge, UK) as previously described [37]. Briefly, 30 μm cryostat sections were blocked with 0.1% Triton X-100 and 10% normal goat serum diluted in 0.1 M phosphate buffer (PB), pH 7.4, for 1 h at room temperature. The primary antibody was added to the blocking solution at a 1:1000 dilution and incubated overnight at 4 °C. Subsequently, sections were washed 5 × 5 min in 0.1 M PB at room temperature, followed by incubation with Alexa Fluor 488-conjugated goat anti-chicken antibody (1:500; Catalogue# A-11039, Invitrogen) in blocking solution for 2 h.

### 2.3 Ca^2+^ imaging and electrophysiology

On the morning of imaging, GCaMP3-PPG mice were deeply anesthetized using isoflurane and decapitated. The brainstem was removed and placed in ice-cold high-Mg^2+^/low-Ca^2+^ artificial cerebrospinal fluid (ACSF; composition in mM: 2.5 KCl, 200 sucrose, 28 NaHCO_3_, 1.25 NaH_2_PO_4_, 7 Glucose, 7 MgCl_2_, 0.5 CaCl_2_; pH 7.4). We cut 200 μm-thick coronal brainstem slices on a vibratome (7000smz2, Campden Instruments) and incubated them in recovery solution (in mM: 3 KCl, 118 NaCl, 25 NaHCO_3_, 1.2 NaH_2_PO_4_, 2.5 Glucose, 7 MgCl_2_, 0.5 CaCl_2_; pH 7.4) at 34 °C for 45 min. Sections were then transferred to standard ACSF (in mM: 3 KCl, 118 NaCl, 25 NaHCO_3_, 10 Glucose, 1 MgCl_2_, 2 CaCl_2_; pH 7.4) and left at 34 °C for a minimum of 30 min before imaging. All solutions were constantly bubbled with 95% O_2_/5% CO_2_.

Electrophysiological recordings in the cell-attached configuration were performed as described previously [42] and analyzed with Strathclyde Electrophysiology Software (WinEDR/WinWCP; University of Strathclyde, Glasgow, United Kingdom). The response firing rate was defined as the average firing rate over 1 min when the firing rate was at its most extreme during the stimulus. Responding cells were identified by determining whether the response firing rate was significantly different to baseline firing rate over 1 min prior to the 5-HT stimulus using Student's T-test. p < 0.05 was taken as statistically significant.

All imaging was performed on PPG neurons in the NTS. Ca^2+^ imaging was performed in one of two configurations: 1) with a Zeiss Axioskop 2FS widefield microscope using a 40× water immersion lens. GCaMP3 was excited using an LED light source (CoolLED pE300white; QImaging) at 460 nm (±25 nm) for 250 ms every 5 s. Images were captured at 12-bit on a charge-coupled device camera (Q-Click; QImaging). 2) with an Olympus FV-1000 scanning confocal microscope using a 25× water immersion lens. GCaMP3 was excited using a laser at 488 nM; emission was recorded at 520 ± 10 nM. Emitted light was detected using a photon-multiplier tube. We observed no difference in the response to 5-HT between the two systems, though bleaching and background fluorescence had to be corrected for only when using the widefield microscope. Slices were continuously superfused with 32 °C standard ACSF at a flow rate of 3–4 ml/min. All drugs were added directly to ACSF. Serotonin hydrochloride (5-HT; 2, 20 and 200 μM), WAY161,503 hydrochloride (5 μM) and TTX-citrate (0.5 μM) were purchased from Tocris Bioscience. 6,7-Dinitroquinoxaline-2,3(1H,4H)-dione (DNQX; 20 μM), 1-Phenylbiguanide (PBG; 1 and 10 μM), granisetron (5 μM), ketanserin (1 μM) and WAY100,635 maleate salt (20 μM) were obtained from Sigma. For recordings in 0 mM Ca^2+^, 2 mM CaCl_2_ was replaced with 2 mM MgCl_2_. Data was collected from at least three different experiments, using tissue derived from at least three mice, for each condition.

### 2.4 Analysis of Ca^2+^ imaging data

Recordings were imported into FIJI image analysis software [43]. XY-drift was adjusted for using the StackReg plugin [44]. Regions of interest (ROIs) and an area for determining background fluorescent intensity were outlined and the mean pixel intensity calculated for each ROI. For time-lapse data recorded on the widefield microscope, background intensity was subtracted from each ROI and recordings were adjusted for bleaching using a cubic polynomial function [45]. Fluorescence intensity data are presented as ΔF/F_0_ with F_0_ being the average fluorescence intensity 5 min prior to the first stimulus and ΔF being the fluorescence intensity, F, minus F_0_. N numbers indicate number of analyzed dendrites. Responses were quantified by calculating areas under the curve (AUC) over 10 min during the stimulus, starting at the beginning of the stimulus. Because they were found not to be normally distributed, summary data are presented as box plots with whiskers, with the median indicated and whiskers marking the 10th and 90th percentile, respectively. Statistical significance was determined using nonparametric statistical testing; Friedman, Kruskal–Wallis or Wilcoxon as indicated in figure legends.

## 3 Results

### 3.1 Serotonergic innervation of PPG neurons

5-HT-immunoreactive innervation of brainstem PPG neurons was assessed in three adult male and three adult female YFP-PPG mice. After peroxidase staining, PPG neurons in the NTS, the IRT and the midline ventral to the hypoglossal nucleus showed intense YFP staining throughout cell bodies and dendrites. The distribution of YFP-expressing neurons in the medulla of YFP-PPG mice was the same as reported before [9], [33], with NTS PPG neurons forming the largest subpopulation and the midline neurons being the smallest.

**Figure 1:**
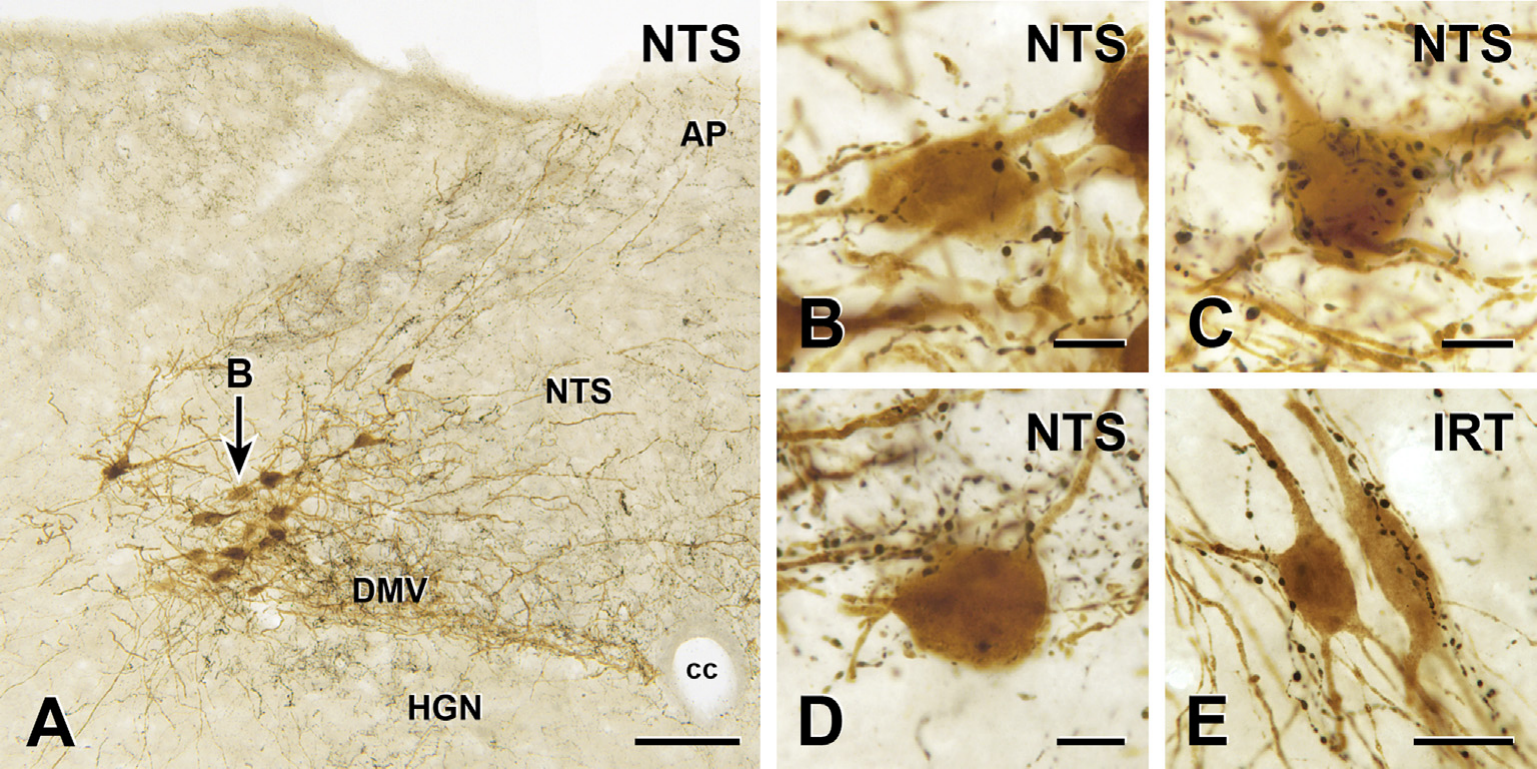
**PPG neurons receive close appositions from 5-HT immunoreactive axons**. Two-color immunoperoxidase labeling for YFP-immunoreactivity in YFP–PPG neurons (brown) and 5-HT-immunoreactivity in serotonergic neurons (black) in transverse sections through the dorsal vagal complex (DVC) of YFP–PPG mice. (A) Montage of low magnification micrographs showing YFP-immunoreactive PPG cell bodies (brown) and 5-HT-immunoreactive axons (black) in the DVC. The cell body indicated by the arrow is shown at higher magnification in B. Scale bar = 100 μm. (B, C, D) Brown YFP-immunoreactive cells bodies and dendrites in the NTS receive close appositions from black 5-HT-immunoreactive axons. Scale bars = 10 μm. (E) A YFP-immunoreactive cell body in the IRT receives close appositions from 5-HT-immunoreactive axons. Scale bar = 20 μm. AP: area postrema; cc: central canal; DMV: dorsal motor nucleus of the vagus; HGN: hypoglossal nucleus; NTS: nucleus tractus solitarius.

**Figure 2:**
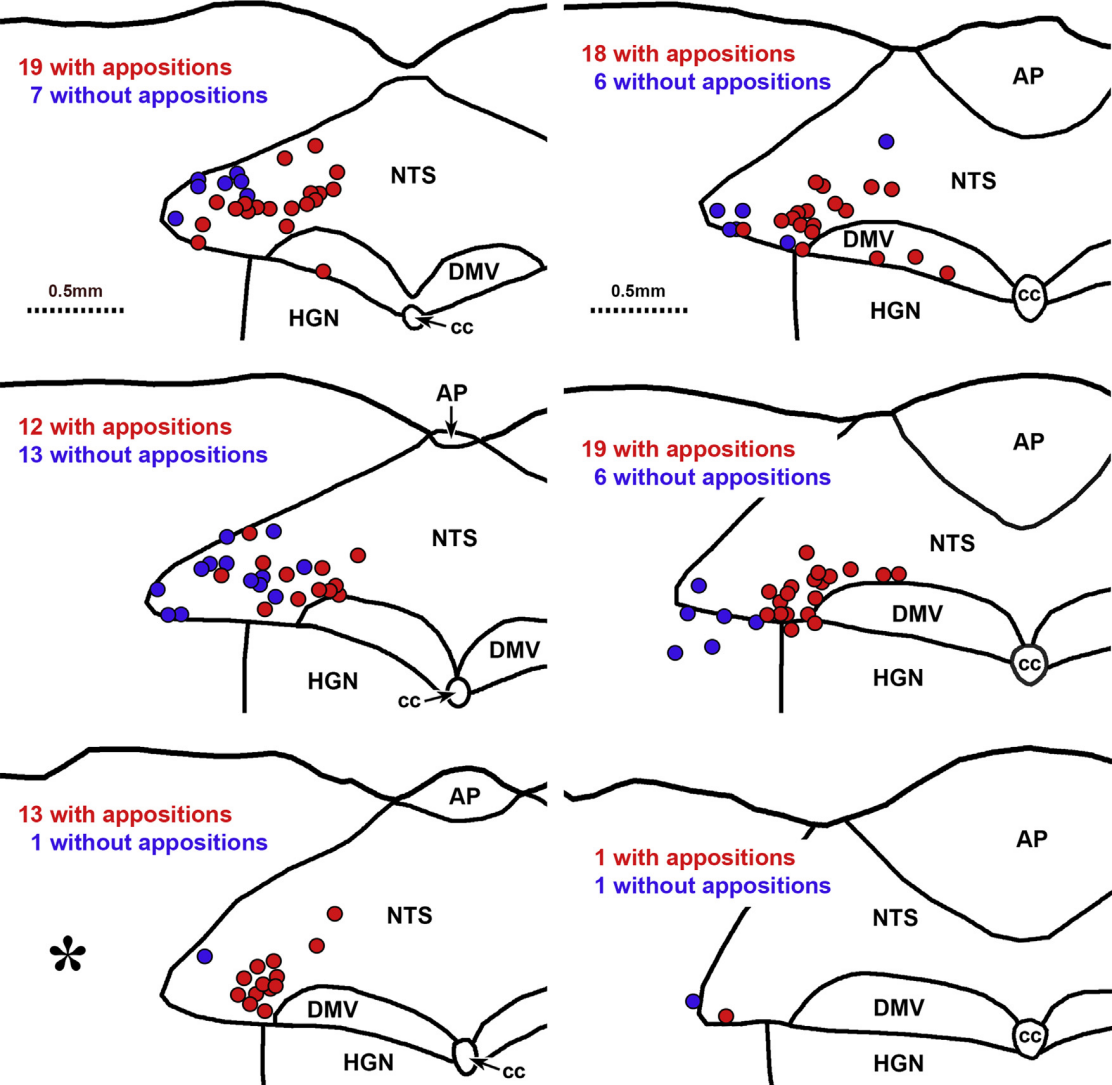
Maps of sections through the dorsal vagal complex of a YFP–PPG mouse showing the distribution of YFP-immunoreactive neurons with and without close appositions from 5-HT-immunoreactive varicosities. Every third section through the brainstem was stained to reveal YFP and 5-HT and then mapped; maps are therefore separated by 60 μm. The most caudal section is at the top left of the figure and the most rostral section is at the bottom right. YFP-immunoreactive neurons are represented by circles. Neurons that receive close appositions from 5-HT-immunoreactive varicosities are shown in red; those that do not receive appositions are shown in blue. Figure 1A shows the section with the asterisk. NTS, nucleus tractus solitarius; AP, area postrema; DMV, dorsal motor nucleus of the vagus; HGN, hypoglossal nucleus; cc, central canal.

5-HT-immunoreactive axons occurred throughout the caudal NTS and many PPG neurons in the NTS received close appositions from 5-HT axons on their somata and/or proximal dendrites (Figure 1A–D). In the NTS, 5-HT axons apposed 208 of 358 PPG neurons (n = 3 mice); the percentage of PPG neurons innervated by 5-HT axons in each mouse varied between 50% and 80%. In each rostro-caudal section analyzed, 5-HT appositions were preferentially targeted toward more medial PPG neurons whereas more lateral neurons usually lacked appositions (Figure 2).

The extent of 5-HT innervation of PPG neurons in the IRT was similar (Figure 1E), with 46% (67 of 147; n = 3 mice) of PPG neurons receiving close appositions. Innervation of midline PPG neurons was very sparse at 4% (2 of 46 PPG neurons with appositions; n = 3 mice).

### 3.2 Intracellular Ca^2+^ can be monitored using GCaMP3

**Figure 3:**
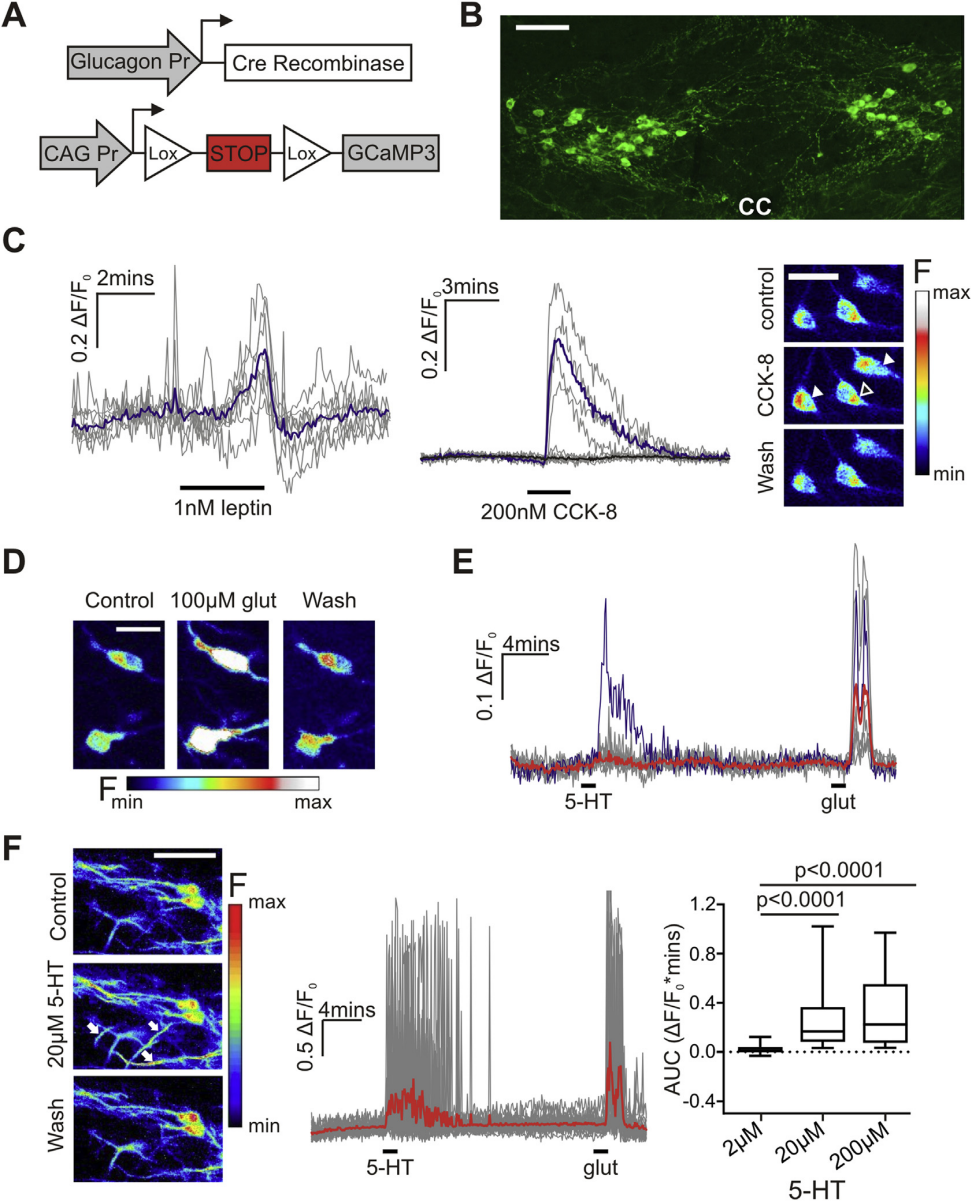
**5-HT evoked Ca^2+^ transients in PPG neurons are mainly observed in dendrites**. (A) Intracellular Ca^2+^ changes in PPG neurons can be monitored using the genetically encoded Ca^2+^ indicator, GCaMP3. Glucagon promoter (Glu)-Cre mice were crossed with mice expressing CAG-promoter-STOP-GCaMP3 in the Rosa26 locus. The Rosa26 locus is active in most cell types. In cells with active glucagon promoter, the bacterial recombinase Cre is produced and excises the STOP sequence flanked by lox sites upstream of the GCaMP3 gene. This process results in cytosolic expression of GCaMP3 in these cells, detected here with an anti-GFP antibody (B; scale bar: 100 μm). (C) PPG neurons respond to both leptin (1 nM, n = 8 somata, left panel) and CCK-8 (200 nM, n = 12 somata, middle panel) with an increase in cytosolic Ca^2+^. The panel on the right shows three pseudocolored cells, two of which (white arrowheads) show increased fluorescence intensity after application of 200 nM CCK-8. The black arrowhead indicates one cell that did not increase [Ca^2+^]_i_ in the presence of 200 nM CCK-8 (Scale bar: 30 μm). (D) GCaMP3 fluorescence intensity is sensitive to changes in cytosolic Ca^2+^ concentration. For example, bath application of 100 μM glutamate (glut) leads to a strong reversible rise in somatic Ca^2+^ concentration (Scale bar: 20 μm). The panel on the left shows two pseudocolored PPG neurons before, during and after stimulation with 100 μM glutamate. (E) Fluorescence intensity expressed as a fraction of the intensity at the beginning of the experiment. Traces from individual cells are plotted in gray or dark blue, and the average response is shown in red. While virtually every cell responded to 100 μM glutamate with a somatic Ca^2+^ transient, in this example, only one of nine PPG neurons responded to 20 μM 5-HT (shown in the dark blue trace). (F) Representative images showing dendritic GCaMP3 fluorescence intensity before, during and after 20 μM 5-HT. Arrows point to dendrites with transient increases in Ca^2+^ (Scale bar = 50 μm). Transient changes in intracellular Ca^2+^ in PPG dendrites (n = 19) in response to 20 μM 5-HT and 100 μM glutamate are shown in the middle. Measurements from individual regions of interest are shown in gray, the mean trace is shown in red. On the right, quantification of responses showing median AUC during the response to 2 μM, 20 μM, or 200 μM 5-HT (n = 88 dendrites (4 mice)). Friedman test (Chi-square = 60.03, p < 0.0001) followed by post-hoc comparisons revealed the response to 2 μM 5-HT to be significantly different to both 20 μM and 200 μM 5-HT (p < 0.0001 for both).

To investigate the potential functional significance of these 5-HT appositions, we recorded intracellular Ca^2+^ changes in NTS PPG neurons in response to 5-HT. Transgenic mice selectively expressing the genetically encoded Ca^2+^ indicator GCaMP3 in PPG neurons (Figure 3A,B) were used to monitor changes in intracellular Ca^2+^ as a surrogate marker for neuronal activity. GCaMP3 was detected in the caudal part of the NTS (Figure 3B) and in the dorsomedial part of the intermediate reticular nucleus (data not shown). GCaMP3-expressing neurons have been shown previously to express GLP-1 in this transgenic mouse model [37]. To demonstrate the ability to record changes in intracellular Ca^2+^ from PPG neurons *in vitro*, coronal brainstem slices were superfused with 100 μM glutamate for 1 min. Intracellular Ca^2+^ rapidly increased in 89% of imaged cell bodies as well as proximal dendrites in response to 100 μM glutamate and returned to baseline after washout of the drug (Figure 3D), demonstrating that rises in intracellular Ca^2+^ are detectable. We have previously shown using patch-clamp electrophysiology that the satiety hormones, leptin and CCK-8, activate PPG neurons in the NTS [34], [35]. Now, using Ca^2+^ imaging, we confirm those findings (Figure 3C). 1 nM leptin increased intracellular Ca^2+^ in all imaged PPG neurons (n = 17 cells), whereas only a subset of NTS PPG neurons exhibited activation upon superfusion with 200 nM CCK-8 (five out of 14 imaged PPG neurons). These data along with previously published immunohistochemical verification [37] validate our model and demonstrate that it is well-suited to investigate responses to other neurotransmitters.

### 3.3 PPG distal dendrites are activated by 5-HT

We next investigated the response of PPG neurons to 5-HT. PPG neurons were exposed to 20 μM 5-HT for 1 min. In the recording shown in Figure 3E, only one cell body responded to 20 μM 5-HT with an increase in intracellular Ca^2+^ (blue trace), although all cell bodies responded to 100 μM glutamate (n = 8 somata). In total, only four out of 39 imaged cell bodies increased intracellular Ca^2+^ in response to 20 μM 5-HT. However, dendrites adjacent to PPG cell bodies responded with rapid, transient rises in intracellular Ca^2+^ (Figure 3F, n = 19 dendrites). The dendritic response was found to be concentration-dependent; 2 μM 5-HT increased dendritic Ca^2+^ spikes to a lesser degree than either 20 μM or 200 μM (Figure 3F right panel, Friedman test, p < 0.0001, n = 88 dendrites (4 mice)). Although cells were only exposed to 5-HT for 1 min, dendritic activity persisted after washout of 5-HT (Figure 3F) and lasted for 3.5 ± 0.2 min.

### 3.4 PPG neurons have functional 5-HT_2_ receptors

**Figure 4:**
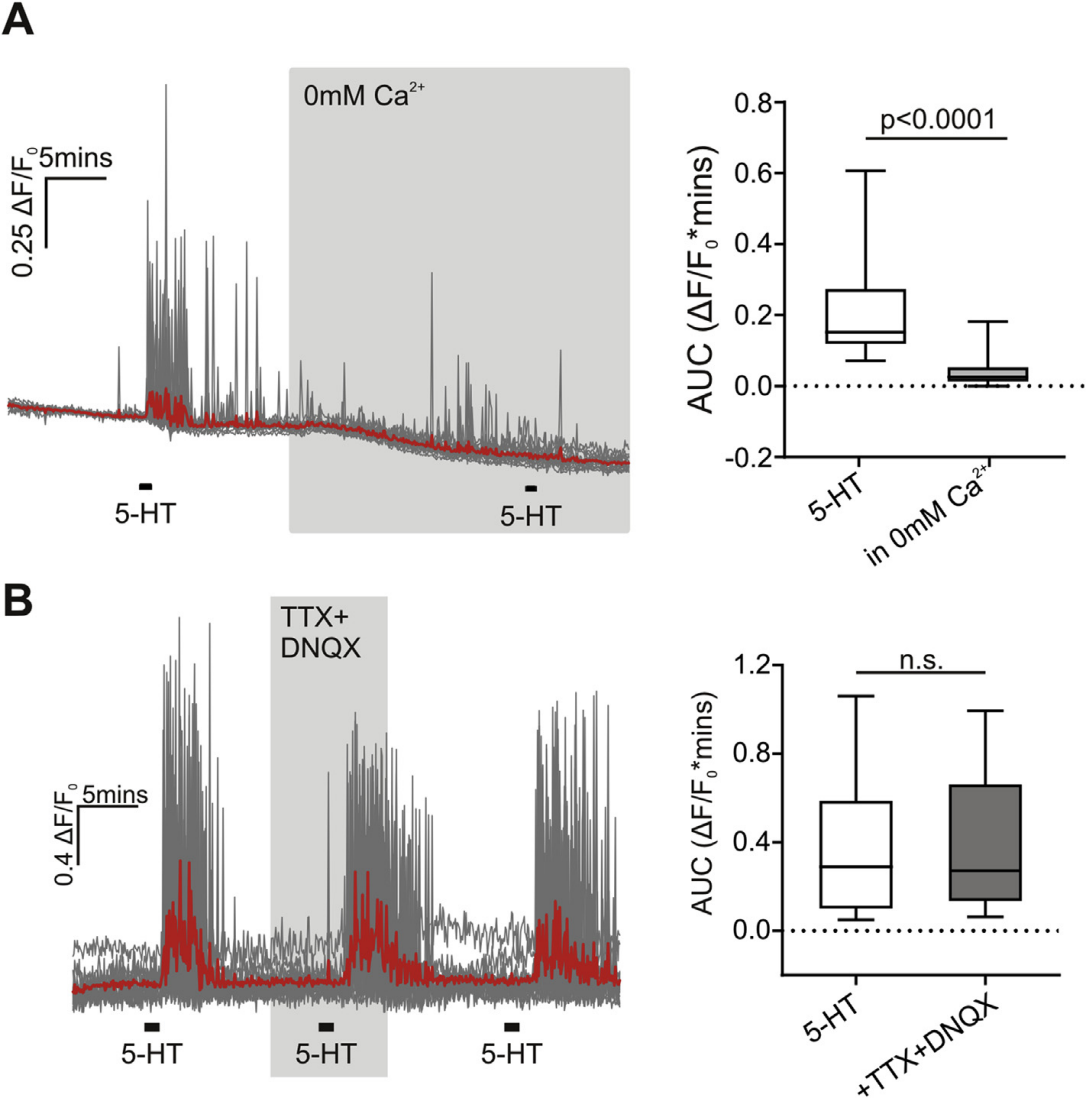
**5-HT activates PPG fibers directly and this is dependent on influx of extracellular Ca^2+^**. (A) Removal of extracellular Ca^2+^ attenuates the response to 20 μM 5-HT. *Left panel*: Traces showing changes in intracellular Ca^2+^ with individual fibers shown in gray and the average trace in red (n = 16 dendrites). *Right panel*: Median AUC in response to 20 μM 5-HT in the presence and absence of extracellular Ca^2+^ (n = 28 dendrites (3 mice); Wilcoxon test: p < 0.0001). (B) *Left panel*: Traces showing the response to 20 μM 5-HT in the presence or absence of TTX (0.5 μM) and DNQX (20 μM) (n = 8 dendrites). *Right panel*: Median AUC during the response to 20 μM 5-HT in the absence (white box) or presence (gray box) of TTX and DNQX (n = 55 dendrites (4 mice)). Wilcoxon test: p = 0.56 n.s.: not significant.

To investigate the source of Ca^2+^ for these responses, we applied 20 μM 5-HT in the absence of extracellular Ca^2+^. Removal of extracellular Ca^2+^ reduced the response to 20 μM 5-HT by 69 ± 12% (Figure 4A, n = 28 (3 mice)). We next asked whether the responses of PPG neurons to 5-HT are dependent on the activity of neighboring cells. By blocking voltage-gated sodium channels with TTX and glutamatergic input with the AMPA/kainate receptor antagonist DNQX, we synaptically isolated the PPG neurons. This assumption is based on our previous observation that blockade of ionotropic non-NMDA receptors inhibits >90% of spontaneous EPSCs in PPG neurons [34], [35]. The response to 5-HT was unaffected by TTX and DNQX, suggesting that PPG neurons have functional 5-HT receptors (Figure 4B, n = 55 dendrites (4 mice)).

**Figure 5:**
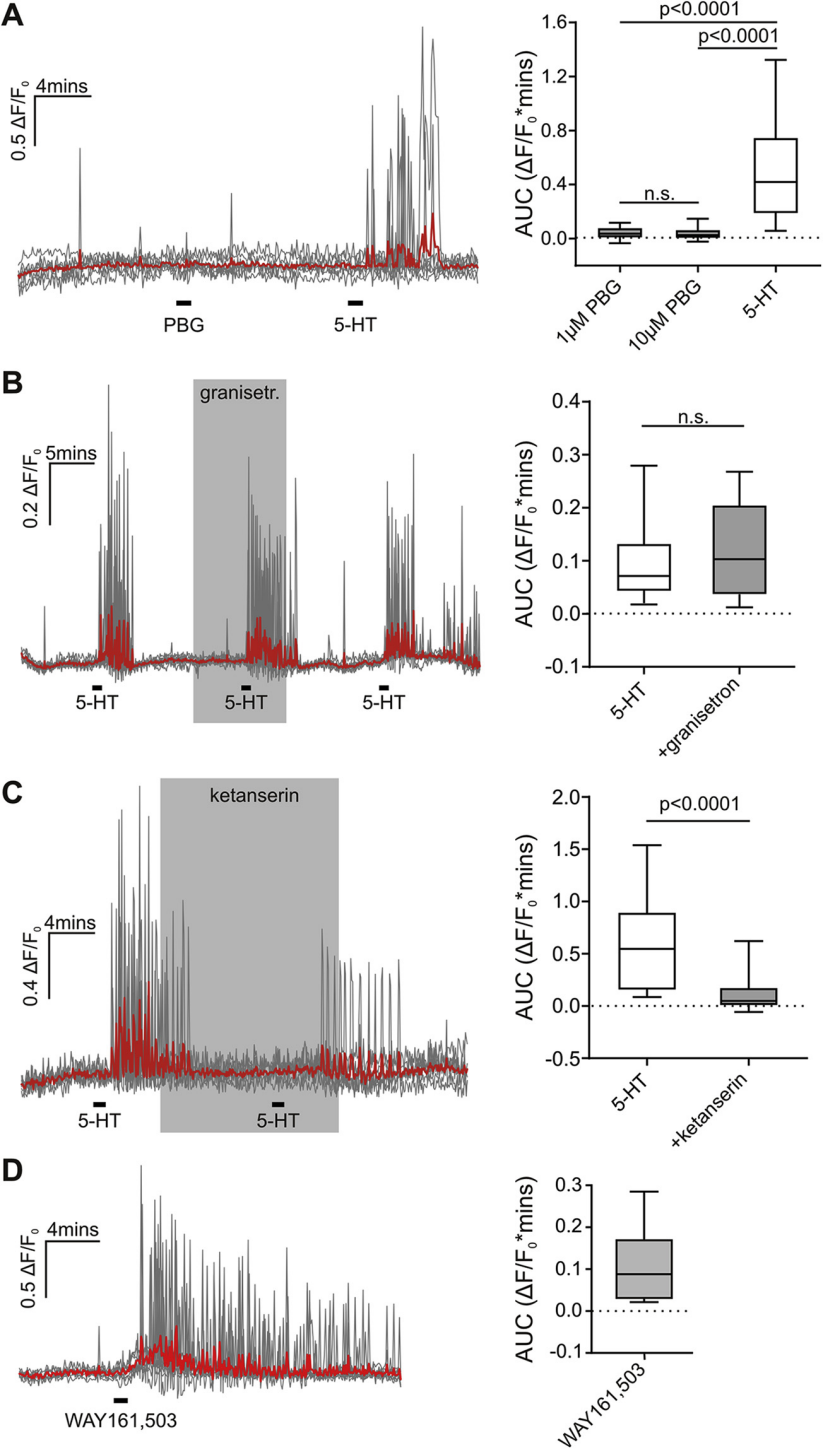
**PPG neurons are activated by 5-HT via 5-HT_2_ but not 5-HT_3_ receptors and respond to 5-HT_2C_ receptor activation with an increase in dendritic Ca^2+^**. (A) *Left panel*: Traces showing the responses to 20 μM 5-HT or 10 μM PBG (n = 7 dendrites), with individual fibers shown in gray and the average trace in red. *Right panel*: Median AUC during the response to 20 μM 5-HT (n = 37 dendrites), 1 μM PBG (n = 32 dendrites) or 10 μM PBG (n = 25 dendrites) (3 mice). Kruskal–Wallis rendered a Chi-Square value of 45.9 (p < 0.0001). Post-hoc comparisons revealed no statistically significant difference between 1 μM and 10 μM PBG (p > 0.999) but showed that the responses to 20 μM 5-HT were significantly different to both 1 μM and 10 μM PBG (p < 0.0001). (B) *Left panel*: Traces showing the response to 20 μM 5-HT in the absence and presence of 5 μM granisetron (granisetr.; n = 6 dendrites). Individual fibers are shown in gray and the average trace in red. *Right panel*: Median AUC for the response to 20 μM 5-HT before (white box) and during (gray box) 5 μM granisetron (n = 32 dendrites (3 mice)). Wilcoxon test: p = 0.12. (C) *Left panel*: Representative traces showing the response to 20 μM 5-HT in the absence and presence of 1 μM ketanserin (n = 6 dendrites). *Right panel*: Median AUC for the response to 20 μM 5-HT in the absence (white box) or presence (gray box) of 1 μM ketanserin (n = 17 dendrites (3 mice)). Wilcoxon test: p < 0.0001. (D) *Left panel*: Representative traces showing the response to 5 μM WAY161,503, a 5-HT_2C_ receptor agonist (n = 9 dendrites). *Right panel*: Median AUC for the response to 1 min 5 μM WAY161,503 (n = 32 dendrites (5 mice)). n.s.: not significant.

Most 5-HT receptor subtypes are expressed in the NTS [46], [47]. 5-HT_2_ and 5-HT_3_ receptor activation is associated with a reduction in food intake; consequently, we assessed the potential contribution of either of these receptors to the response to 5-HT in PPG neurons. The 5-HT_3_ receptor agonist phenylbiguanide (PBG) failed to elicit Ca^2+^ changes at two different concentrations (Figure 5A; 1 μM PBG, n = 22 dendrites and 10 μM PBG, n = 25 dendrites; 20 μM 5-HT, n = 37 dendrites (3 mice)) and 5 μM granisetron, a 5-HT_3_ receptor antagonist, was unable to block the response to 20 μM 5-HT (Figure 5B, n = 32 dendrites (3 mice), p = 0.12). These findings suggest that 5-HT_3_ receptors are not involved in the response of PPG neurons to 5-HT. In contrast, blocking 5-HT_2_ receptors with 1 μM ketanserin attenuated the response to 5-HT by 77 ± 6% (Figure 5C, n = 17 dendrites (3 mice)).

Next we tested whether the 5-HT_2C_ receptor agonist WAY161,503 is able to elicit dendritic Ca^2+^ spikes in PPG neurons because 5-HT_2C_ receptors are well known to be involved in the regulation of food intake. WAY161,503 (5 μM) elicited transient rises in Ca^2+^ in PPG dendrites in a total of five different sections from five different animals (Figure 5D, n = 32 dendrites (5 mice)). This response to 5-HT_2C_ receptor activation suggests that 5-HT activates PPG neuronal dendrites via 5-HT_2C_ receptors.

### 3.5 5-HT modulates spontaneous firing activity of PPG neurons

**Figure 6:**
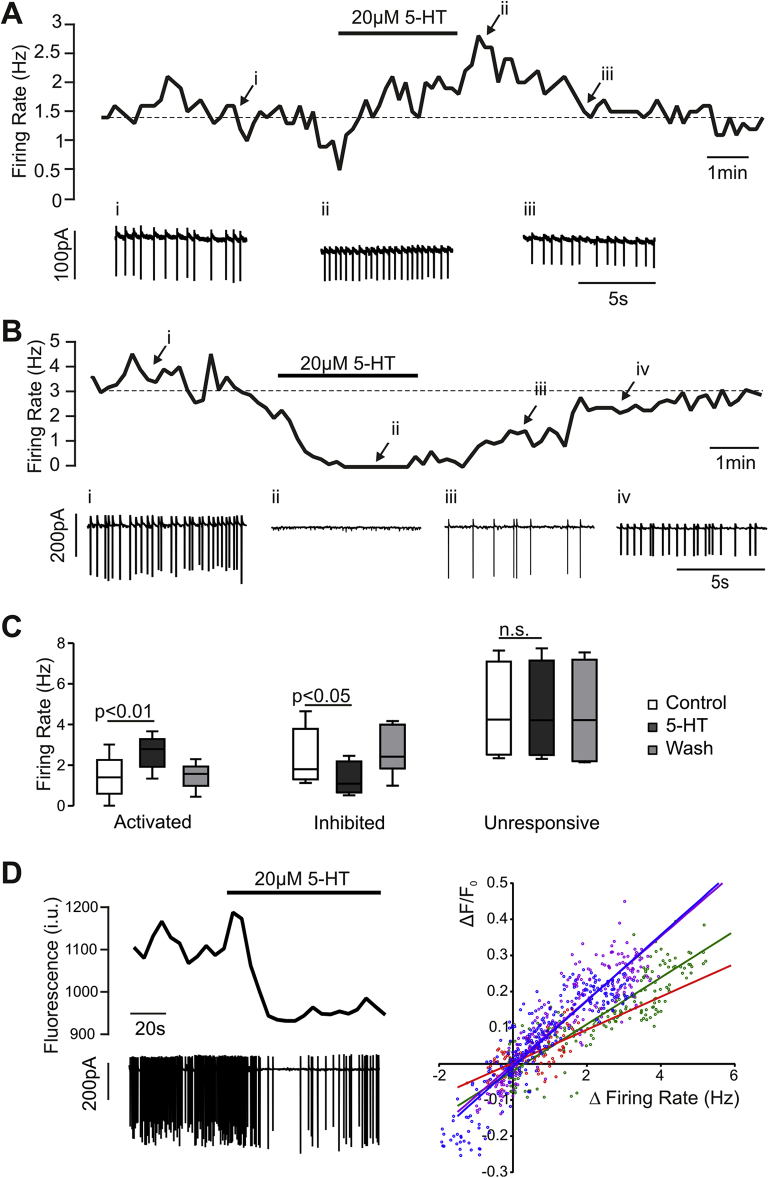
**5-HT modulates the electrical activity of PPG neurons**. (A,B) Spontaneous firing rate recorded from PPG neurons in loose-patch cell-attached configuration. Periods of the original recordings at time points i, ii, iii, and iv are displayed below the graphs of instantaneous firing rate. Some PPG neurons are activated by 5-HT (A), whereas others are inhibited (B) or do not change firing rate (not shown). (C) Box and whiskers plot of data from cells that were activated (n = 5 cells) or inhibited (n = 7 cells) by 5-HT and from cells that did not change their firing rate in response to 5-HT (n = 4) (data from 8 mice). A one-way within subjects ANOVA revealed a significant effect of 5-HT in the activated (p < 0.01) and inhibited (p < 0.01) cells but not in the unresponsive cells. A post-hoc analysis (Tukey) revealed a statistically significant increase in firing rate compared to baseline in the activated cells (p < 0.01) and a decrease in the inhibited cells (p < 0.05). (D) *Left panel*: Simultaneous recording of firing activity in attached patch configuration (bottom trace) and of intracellular Ca^2+^ (top trace, i.u.: intensity units) revealed a positive correlation between firing rate and intracellular Ca^2+^ concentration. The black bar indicates application of 20 μM 5-HT. *Right panel*: Correlation between changes in firing rate and intracellular Ca^2+^ levels for four individual cells analyzed. Each cell is represented by a unique color, individual data points are represented by dots and a linear fit to the data is plotted in the same color (data from 3 mice).

The results above demonstrate that PPG neurons receive 5-HT synaptic inputs and that activation of 5-HT_2C_ receptors modulates intracellular Ca^2+^ concentration in PPG dendrites *in vitro*. Next, we explored whether these dendritic changes in intracellular Ca^2+^ reflect a change in the electrical output from these cells. We used extracellular recordings in the loose-patch configuration to monitor the pattern of spontaneous action potential discharges in PPG neurons without breaking the cell membrane and diluting cytosolic proteins. This approach enabled us to record electrical activity and cytosolic Ca^2+^ simultaneously, because GCaMP3 was not diluted into the patch-pipette. Of the 16 cells recorded, seven reduced their firing rate in the presence of 20 μM 5-HT (Figure 6B); four cells showed no response and five increased their firing rate (Figure 6A,C). A significant reduction in firing rate was accompanied by a small, but clear reduction in intracellular Ca^2+^ concentration (Figure 6D), demonstrating that ongoing electrical activity strongly affects the intracellular Ca^2+^ concentration in PPG neurons. This correlation between firing rate and intracellular Ca^2+^ concentration is further demonstrated in Figure 6D, right panel.

**Figure 7:**
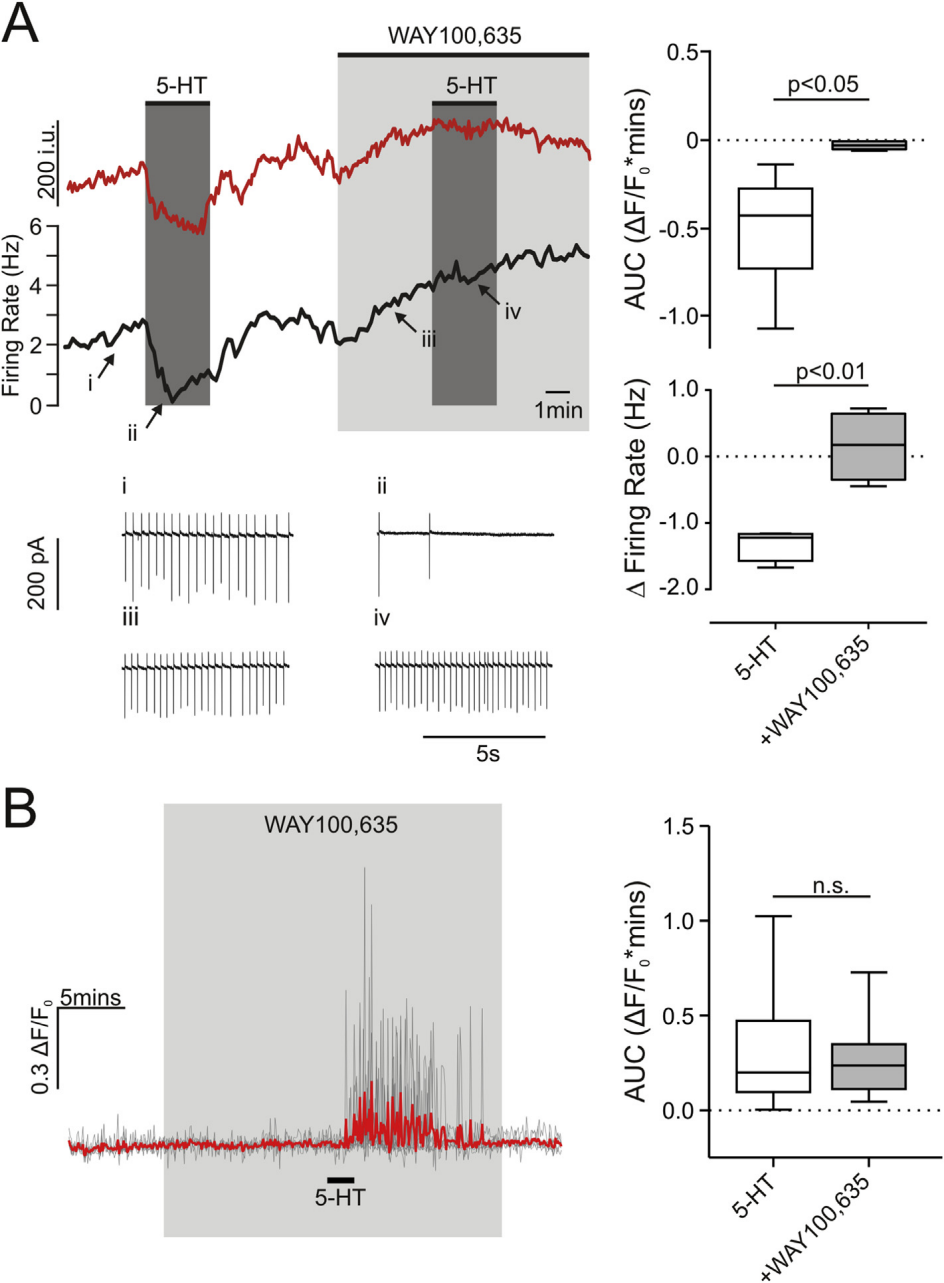
**5-HT inhibition of PPG neuron firing rate is dependent on 5-HT_1A_ receptors**. (A) Change in GCaMP3 fluorescence (top left panel, i.u.: intensity units) and spontaneous firing rate (middle panel) from a PPG neuron recorded in the loose-patch cell-attached configuration. Applications of 20 μM 5-HT are indicated by dark gray boxes and application of 5-HT_1A_ receptor antagonist, WAY100,635 (20 μM), by a light gray box. Raw recordings at time points i, ii, iii, iv are shown in the bottom left panel. *Bottom right panel*: Summary data comparing the change in firing rate in response to 5-HT alone and in the presence of WAY100,635 (n = 4 cells (4 mice)). Paired Student's t-test: p < 0.01. *Top right panel*: Ca^2+^ recordings from seven somata. Intracellular Ca^2+^ decreased in response to 5-HT. This inhibitory effect of 5-HT was absent in the presence of WAY100,635 (n = 7 somata (3 mice)). Wilcoxon test: p < 0.05. (B) *Left panel*: The dendritic response to 5-HT was unchanged in the presence of 20 μM WAY100,635 (n = 6 dendrites). The black bar indicates application of 20 μM 5-HT. The light gray box indicates WAY100,635 application. *Right panel*: Summary data comparing 5-HT alone from previous experiments (n = 248 dendrites) with 5-HT in the presence of WAY100,635 (n = 20 dendrites (3 mice)). n.s.: not significant (p = 0.91) according to Mann–Whitney test.

Having established that 5-HT reduces firing rate in a proportion of PPG neurons, we next addressed the question which 5-HT receptors are involved in this response. Only 5-HT_1_ and 5-HT_5_ receptors couple to the G_i/o_ pathway [48] and 5-HT_1A_ receptor inhibition reduces food intake [27]. We therefore tested in four PPG neurons that decreased firing rate under 5-HT whether the selective 5-HT_1A_ receptor antagonist WAY-100,635 reduces the inhibitory effect, and whether 5-HT_1A_ receptor inhibition facilitates somatic Ca^2+^ rises under 5-HT by blocking inhibitory responses. Figure 7A shows a representative loose-patch recording from a PPG neuron that was strongly inhibited by 5-HT (left) along with the change in fluorescence intensity of the same cell (top left; red trace). Action potential firing ceased almost completely upon application of 20 μM 5-HT. In the presence of WAY100,635, the inhibitory response to 5-HT was prevented as documented in both the Ca^2+^ imaging trace and the firing frequency but did not turn into a 5-HT-mediated excitation in any of the four cells tested (bottom right). Somatic Ca^2+^ recordings produced a similar result with seven cells that showed a clear reduction in intracellular Ca^2+^, losing that inhibitory response in the presence of WAY100,635 (Figure 7A top right). Interestingly, the dendritic response to 20 μM 5-HT was not altered in the presence of WAY100,635 (Figure 7B: left panel, n = 6; right panel, 5-HT, n = 248; right panel, +WAY100,635, n = 20 (3 mice)), demonstrating that blocking inhibitory 5-HT receptors does not increase excitation.

## 4 Discussion

In this study, we explored potential anatomical and functional links between 5-HT and GLP-1 neurons within the brainstem. First, we demonstrated that GLP-1-producing PPG neurons in the medulla receive close appositions from 5-HT-immunoreactive axons. We found close appositions from serotonergic axons on the somata or proximal dendrites of 58% of PPG neurons in the NTS and lower levels of innervation of the PPG neurons in the IRT. Interestingly, midline PPG neurons received very sparse 5-HT input, suggesting these neurons could play a different physiological role to the NTS and IRT populations.

Despite almost two thirds of NTS PPG neurons receiving somatic or proximal dendritic close appositions, only 10% of these cells exhibited a somatic rise in intracellular Ca^2+^ upon exposure to 5-HT and approximately a quarter of PPG neurons increased their electrical activity in response to 5-HT. However, we also observed that 5-HT application reduced electrical activity in almost 50% of the NTS PPG neurons tested. This finding suggests that most of the axosomatic close appositions from 5-HT terminals onto PPG neurons reflect inhibitory inputs.

The majority of Ca^2+^ increases in response to 5-HT were recorded in dendrites with little observable propagation of the Ca^2+^ signal to cell bodies. We were therefore unable to quantify the percentage of PPG neurons that were excited by 5-HT at dendritic level. We found that dendritic Ca^2+^ rises evoked by 5-HT persisted in the presence of TTX and DNQX. These observations support the notion that the dendritic responses are due to activation of postsynaptic 5-HT receptors rather than presynaptic receptors that increase glutamate release onto PPG neurons, as we observed for CCK [34] and as was reported for 5-HT on A2 catecholaminergic NTS neurons [49].

### 4.1 Excitatory 5-HT responses

5-HT_3_ receptors are strongly expressed on vagal afferent terminals in the NTS, and we have previously demonstrated that PPG neurons receive both mono- and polysynaptic inputs from vagal afferents [35]. Interestingly, however, neither selective activation of 5-HT_3_ receptors nor selective inhibition of these receptors during 5-HT application significantly changed Ca^2+^ concentrations in PPG neurons. This result argues that PPG neurons do not express 5-HT_3_ receptors and suggests that vagal afferents expressing 5-HT_3_ receptors do not impinge upon these cells. However, it should be noted that we cannot categorically exclude that a 5-HT-induced increase in glutamatergic postsynaptic potentials from vagal afferents may fail to affect intracellular Ca^2+^ in PPG neurons sufficiently to be detected using GCaMP3. Nevertheless, activation of any presynaptic 5-HT_3_ receptors should have measurably affected Ca^2+^ concentrations in PPG neurons since our combined loose-patch recordings and our Ca^2+^ recordings showed a close correlation between Ca^2+^ levels and electrical activity. Our finding that PPG neurons do not express 5-HT_3_ receptors is consistent with the observation by Appleyard and colleagues that, while the vast majority of A2/C2 neurons in the NTS respond to 5-HT_3_ receptor activation, only a small fraction of non-catecholaminergic cells do [49]. 5-HT released from serotonergic axons in the NTS acts on 5-HT_3_ receptors to provide a strong anorexic drive to the parabrachial nucleus (PBN) [50]. Because our results demonstrate that PPG neurons do not have functional 5-HT_3_ receptors, we conclude that PPG neurons do not directly contribute to the NTS – PBN projection underlying this hypophagic drive.

The 5-HT input to PPG neurons identified here seems to act on 5-HT_2_ rather than 5-HT_3_ receptors. 5-HT_2C_ receptors have been implicated in the control of food intake via the melanocortin system in the hypothalamus [51]. We show here that 5-HT-induced dendritic Ca^2+^ spikes are dependent on 5-HT_2_ receptors and that activation of 5-HT_2C_ receptors increases dendritic Ca^2+^ in NTS PPG neurons. Whether activation of 5-HT_2C_ receptors on PPG neurons contributes to the anorexic effects of, for example, the 5-HT_2C_ receptor agonist lorcaserin remains to be determined. Whilst the anorexic 5-HT receptor agonist m-chloro-phenylpiperazine (mCPP) failed to elicit Fos-immunoreactivity in rat PPG neurons [52], a study using 5-HT_2C_ knockout mice reported that the satiating effect of GLP-1 is blunted in the absence of 5-HT_2C_ receptors [32]. However, the knockout study links GLP-1 receptor activation and 5-HT_2C_ receptors, not specifically 5-HT_2C_ reception on PPG neurons. Furthermore, even if PPG neurons are involved, the effect of the loss of 5-HT_2C_ receptors may reside downstream, rather than upstream, from the PPG neurons.

The results discussed so far suggest an excitatory role for 5-HT_2C_ receptors on dendrites and a lack of propagation of the Ca^2+^ signal to somata, an effect that has been described previously for other central neurons, including neocortical and hippocampal pyramidal neurons [53], [54]. A response to 5-HT that is confined to a specific subcellular compartment could have several causes. We reject the hypothesis that 20 μM 5-HT could be subthreshold for activation of PPG neurons *in vitro* because increasing the concentration of 5-HT to 200 μM failed to elicit somatic responses and had no further effect on dendritic Ca^2+^ spiking. Alternatively, activation of 5-HT_2C_ receptors on dendrites would result in depolarization locally but failure of the signal to reach the soma due to simultaneous activation of inhibitory 5-HT receptors on the cell body. However, we can reject this scenario as well because we should have observed somatic Ca^2+^ increases upon selective activation of 5-HT_2C_ receptors using WAY161,503. In fact, 5-HT_2C_ receptor activation mimicked the response to 5-HT. We therefore propose that activation of 5-HT_2C_ receptors on PPG neurons leads to compartmentalized increases in Ca^2+^ in dendrites but not somata. Such local Ca^2+^ transients have previously been implicated in synaptic plasticity, enhancing somatic spike precision and dendritic release of neurotransmitters, including neuropeptides [53].

### 4.2 Inhibitory responses to 5-HT

Although optical recordings mainly revealed effects of 5-HT on dendritic Ca^2+^ spikes, our immunohistochemical results provide evidence for close appositions from 5-HT-containing axons not only onto distal dendrites but also onto somata and proximal dendrites. These observations suggest that PPG neurons are able to respond to 5-HT somatically in addition to the dendritic 5-HT_2C_ receptor-mediated Ca^2+^ spikes that we observed with optical recordings. With that possibility in mind, we performed cell-attached patch clamp recordings to explore both excitatory and inhibitory changes in firing frequency of spontaneously active PPG neurons upon exposure to 5-HT. The cell-attached firing rate was similar to that observed in perforated whole cell recordings in our earlier studies and changes in firing rate correlated with changes in intracellular Ca^2+^ [34], [35]. Only a quarter of electrically recorded cells increased firing rate in response to 5-HT, in accordance with our finding that few PPG neurons showed an increase in somatic Ca^2+^. More significantly, we observed that 5-HT application reduced electrical activity in almost half of the NTS PPG neurons tested. 5-HT-induced inhibition of firing rate was dependent on 5-HT_1A_ receptors that presumably occur on somata.

Interestingly, blocking the inhibitory response to 5-HT with WAY100,635 did not lead to excitation of PPG neurons by 5-HT. This finding has two possible explanations, which are not mutually exclusive. Firstly, the PPG neurons that exhibit dendritic calcium rises and the neurons in which somata are inhibited may belong to separate populations. Secondly, the pronounced dendritic activation observed in the presence of 5-HT does not lead to somatic and axonal activation, even when inhibitory 5-HT responses at the soma are blocked, because the dendritic compartment is functionally isolated from the soma, at least in respect to Ca^2+^ spikes. While we cannot determine whether the excitatory and inhibitory 5-HT responses occur on separate PPG neuron populations, the failure of the 5-HT_2C_ receptor agonist to produce somatic excitation, discussed in the previous section, strongly argues for compartmentalization of the dendritic calcium spikes.

### 4.3 Physiological significance

Our study has shown that 5-HT has two main effects on PPG neurons. First, it inhibits the electrical activity of approximately 50% of NTS PPG neurons via 5-HT_1A_ receptors. At the same time, 5-HT also produces significant activation of the dendritic compartment of PPG neurons via 5-HT_2C_ receptors. Both 5-HT_1A_ receptors and 5-HT_2C_ receptors have been implicated in the regulation of food intake. 5-HT_1A_ receptor blockade suppresses food intake [27], as disinhibition of PPG neurons would do. In addition, loss of 5-HT_2C_ receptors produces hyperphagia [30], suggesting that 5-HT_2C_ receptor activation reduces appetite, as has been demonstrated for activation of PPG neurons [15]. Thus, the opposite effects of activation of these two types of 5-HT receptors on PPG neurons align with the physiological roles of these receptors.

It was beyond the scope of this study to determine the origin of the 5-HT-immunoreactive axons that appose PPG neurons. However, serotonergic axons from more than one CNS site, including the raphe magnus and obscurus but not the dorsal raphe nuclei, converge in the NTS [50], [55]. A detailed anatomical dissection of the origin of individual serotonergic inputs may help explain the heterogeneous response to 5-HT observed in the current study and further clarify how serotonin modulates the activity of PPG neurons.

## 5 Conclusions

In the brain, both GLP-1 and 5-HT control a wide range of tightly-regulated homeostatic processes, including food intake and cardiovascular function. We have previously found that PPG neurons innervate 5-HT-containing neurons in the caudal raphe and show here that many PPG neurons in turn receive serotonergic innervation. However, cellular responses to 5-HT in PPG neurons were heterogeneous and compartmentalized. The firing rates of some PPG neurons increased upon 5-HT application while the firing rates of other PPG neurons decreased. In addition, distal dendrites showed transient Ca^2+^ spikes that failed to propagate to the soma. Identification of the 5-HT receptor types involved in these opposite responses, revealed that these receptors are also reported to have opposite effects on food intake, thus conforming to a role of GLP-1 as a satiety factor. The data reported here indicate that within the brain there are complex links between 5-HT and GLP-1 transmission, which are likely to be important for the regulation of satiety.

## Author contributions

ST and ILS conceived the project. FR and FMG provided the transgenic mice. MKH performed the *in vitro* experiments. ILS performed the immunohistochemistry. ST and MKH wrote the manuscript and all authors contributed to editing and provided intellectual input.
